# Evolution of an adaptive behavior and its sensory receptors promotes eye regression in blind cavefish

**DOI:** 10.1186/1741-7007-10-108

**Published:** 2012-12-27

**Authors:** Masato Yoshizawa, Yoshiyuki Yamamoto, Kelly E O'Quin, William R Jeffery

**Affiliations:** 1 Department of Biology, University of Maryland, College Park, MD 20742, USA; 2 Department of Cell and Developmental Biology, University College London, Gower Street, London WC1E 6BT, UK

**Keywords:** animal behavior, regressive evolution, constructive evolution, neuromast, hedgehog, tradeoff, quantitative trait locus, eye, QTL cluster, adaptation

## Abstract

**Background:**

How and why animals lose eyesight during adaptation to the dark and food-limited cave environment has puzzled biologists since the time of Darwin. More recently, several different adaptive hypotheses have been proposed to explain eye degeneration based on studies in the teleost *Astyanax mexicanus*, which consists of blind cave-dwelling (cavefish) and sighted surface-dwelling (surface fish) forms. One of these hypotheses is that eye regression is the result of indirect selection for constructive characters that are negatively linked to eye development through the pleiotropic effects of Sonic Hedgehog (SHH) signaling. However, subsequent genetic analyses suggested that other mechanisms also contribute to eye regression in *Astyanax *cavefish. Here, we introduce a new approach to this problem by investigating the phenotypic and genetic relationships between a suite of non-visual constructive traits and eye regression.

**Results:**

Using quantitative genetic analysis of crosses between surface fish, the Pachón cavefish population and their hybrid progeny, we show that the adaptive vibration attraction behavior (VAB) and its sensory receptors, superficial neuromasts (SN) specifically found within the cavefish eye orbit (EO), are genetically correlated with reduced eye size. The quantitative trait loci (QTL) for these three traits form two clusters of congruent or overlapping QTL on *Astyanax *linkage groups (LG) 2 and 17, but not at the *shh *locus on LG 13. Ablation of EO SN in cavefish demonstrated a major role for these sensory receptors in VAB expression. Furthermore, experimental induction of eye regression in surface fish via *shh *overexpression showed that the absence of eyes was insufficient to promote the appearance of VAB or EO SN.

**Conclusions:**

We conclude that natural selection for the enhancement of VAB and EO SN indirectly promotes eye regression in the Pachón cavefish population through an antagonistic relationship involving genetic linkage or pleiotropy among the genetic factors underlying these traits. This study demonstrates a trade-off between the evolution of a non-visual sensory system and eye regression during the adaptive evolution of *Astyanax *to the cave environment.

## Background

The dark and nutrient poor cave environment exerts substantial pressure upon cave-dwelling animals. Perhaps as a consequence of these limited resources, cave-adapted animals from most major phyla exhibit a remarkable convergence in morphological and physiological changes related to cave life, including features that are both constructive (the lengthening of legs, fins and antennae, the appearance of novel behaviors, and elaboration of non-visual sensory systems) and regressive (the reduction or loss of vision and pigmentation) [1,2]. Although these changes have been studied in a diverse set of cave animals, the genetic and evolutionary mechanisms responsible for them remain poorly understood.

Although natural selection is probably involved in the evolution of constructive cave adapted phenotypes [3], the evolutionary forces driving regressive changes are less certain. Darwin suggested that eye degeneration - one of the most conspicuous traits found in cave animals - evolved due to disuse [4]. This idea was refined by others to implicate neutral mutation and genetic drift as a consequence of relaxed selection for vision in the cave environment [5,6]. The neutral hypothesis was favored until recently when the results of new genetic and developmental studies supported the adaptive evolution of eye regression in cave animals. Three competing hypotheses have been proposed for the adaptive evolution of eye regression: (1) direct natural selection against eyes to conserve energy in the resource poor cave environment [7]; (2) indirect selection against eyes to open sufficient space for the elaboration of constructive characters [2,8-10]; and (3) indirect selection against eyes due to the enhancement of traits that are negatively linked to optic development by antagonistic pleiotropy [11,12]. Distinguishing among these hypotheses has been difficult since the genetic basis of eye reduction is unknown in cave-dwelling animals.

The teleost *Astyanax mexicanus *is an excellent model organism for studying the evolution of traits associated with cave life, including eye regression [5,13-16]. Within the past few million years, at least five independent colonizations by two different migrational waves of eyed surface fish have established 29 geographically isolated *Astyanax *cavefish populations in northeastern Mexico [17-20]. After subsequent radiation underground, the founder cavefish populations became isolated in separate caves and evolved eye regression, reduced pigmentation or albinism, enhanced sensory systems and behavioral changes associated with cave life [5,11,12,21-32]. Despite this isolation, *Astyanax *surface fish and cavefish are interfertile in the laboratory, allowing the evolution of constructive and regressive traits to be studied by genetic analysis.

Previous studies reported that eye degeneration in *Astyanax *is triggered by lens apoptosis and dysfunction due to expanded *sonic hedgehog *(*shh*) gene expression along the embryonic midline [25,26]. Additionally, *shh *hyper expression was shown to increase jaw width and taste bud number, and to mediate the expansion of the forebrain and hypothalamus [11,22]. In a recent study, Elipot *et al. *found that *shh *modifies the hypothalamic serotonergic network and increases foraging efficiency in cavefish by shifting behavior from fighting to foraging [32]. These experiments support the hypothesis that eye regression in *Astyanax *has evolved at least in part as a result of indirect selection against eyes in favor of increased feeding efficiency through pleiotropy of the *shh *genes. However, recent genetic studies have discovered 8 to 12 quantitative trait loci (QTL) involved in eye reduction in *Astyanax *cavefish [5,7,33], but none that are linked to either the *shhA *or *shhB *genes, suggesting that upstream modulators of the SHH signaling system and/or other genetic factor(s) may be important in eye regression (for review, see ref. [3]).

In the present study, we launch an alternative approach to address the evolutionary mechanisms involved in eye degeneration. Assuming a positive relationship between visual decay and the evolution of constructive changes in other sensory systems - as has been proposed by many previous investigators [2,8,10,34,35] - we investigated the genetic basis of a constructive trait, the vibration attraction behavior (VAB), to evaluate its possible relationship to eye degeneration. VAB is the swimming of cavefish toward an oscillating object, a behavior that has evolved repeatedly in different *Astyanax *cavefish populations [27,36,37] and which may be present in Amblyopsid cavefish as well [38,39]. VAB is mediated by an increase in the number and size of cranial superficial neuromasts (SN) in cavefish [27]. Although VAB is usually absent in surface fish, a small proportion of those raised in the laboratory can show a weak form of VAB. In wild populations of surface fish, VAB is presumably deleterious since it may be easily detectable by predators [40]. In contrast, VAB is adaptive in cavefish since it increases foraging in an environment devoid of light with sparse food and no macroscopic predators [27,40].

Here we report the results of genetic analyses of VAB, SN enhancement and eye size. Using this approach, we discovered that the QTL underlying VAB, a specific class of SN located within the cavefish eye orbit (EO), and reduced eyes form two distinct clusters of overlapping QTL that together explain a significant portion of the genetic variation underlying these traits. By ablation of EO SN, we discovered that these sensory receptors contribute to VAB. Moreover, by *shh *overexpression, we also showed that induction of eye degeneration in surface fish did not induce VAB or increase EO SN, suggesting that these traits are part of an antagonistic system impacting eye formation that is independent of SHH signaling, and that the extra space opened by eye regression is insufficient to promote the appearance of these constructive traits. Therefore, we propose that the adaptive evolution of VAB and EO SN enhancement has contributed to eye degeneration in *Astyanax *cavefish.

## Results

### A small number of genetic factors control VAB and SN number

**Figure 1:**
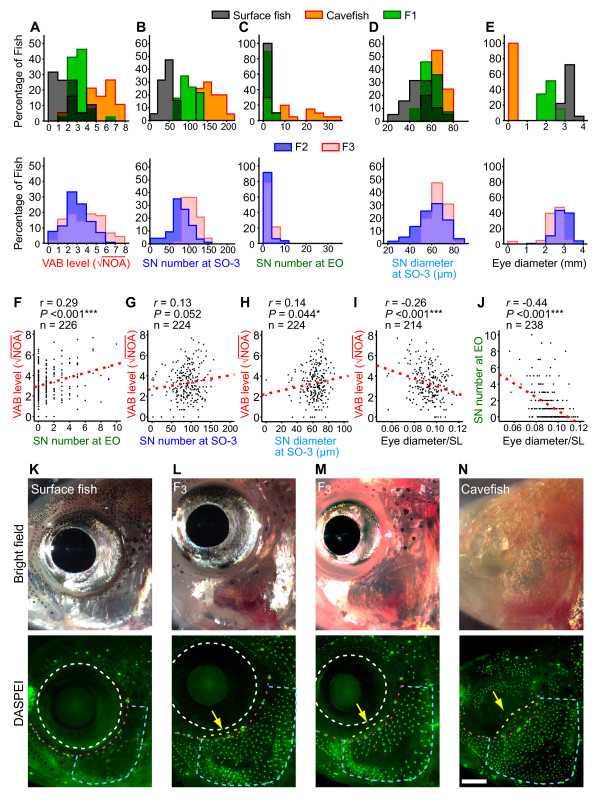
**Genetic analysis of VAB, SN and eye regression**. (**A-E**) Histograms showing (A) VAB level (square-rooted number of approaches, or NOA), (B) SO-3 SN number, (C) EO SN number, (D) SO-3 SN diameter, and (E) eye diameter in surface fish, cavefish and their F_1 _progeny (upper frames), and the F_2 _and F_3 _generations (lower frames). The F_1 _and F_2 _phenotypes are intermediate between surface fish and cavefish, and the sum of F_2 _and F_3 _phenotypes covered most of the range of phenotypes for each trait between surface fish and cavefish, although the distributions of SO-3 and EO SN numbers remain somewhat restricted towards the surface fish phenotype. (**F-G**) Regression analysis showing the relationships between VAB and (F) EO SN number, (G) SO-3 SN number, (H) SO-3 SN diameter, and (I) eye diameter/SL. (J) The relationship of SN number at EO and eye diameter/SL. EO SN number and SO-3 SN diameter were both positively correlated with VAB level, which was negatively correlated with eye size. EO SN number was also negatively correlated with eye size. Linear regression lines are shown in red. (**K-N**) Bright field images (upper) and DASPEI-stained neuromasts (lower) compared among (K) surface fish, (L-M) two examples of F_3 _hybrids, and (N) cavefish. Scale bar in (N) is equal to 1.0 mm. In K-N, circles outlined by white dashed lines indicate the edges of the eye, red dashed lines indicate the lines of suborbital canal neuromasts in the head lateral line, the areas enclosed by the blue dotted lines indicate the approximate outline of the SO-3 region, and the areas shown by the yellow dotted lines and indicated by yellow arrows show the EO regions.

To determine the phenotypic and genetic relationship among eye size, VAB and SN enhancement, we first estimated the number of genetic factors that control these traits. We crossed a female Texas surface fish with a male Pachón cavefish and raised about 130 F_1 _individuals. One pair of these F_1 _progeny was crossed to generate 384 F_2 _individuals for phenotypic and genetic analysis. We then measured the degree of VAB, SN number and size at suborbital bone 3 (SO-3 SN) [41], SN number within the eye orbit (EO) and overall eye size in each individual. The distribution of VAB and SN scores among the P_0_, F_1 _and F_2 _generations suggests that all four traits are heritable and controlled by one or more genetic factors (Figure 1A-D). Classic Castle-Wright estimates of the effective number of loci for VAB, SO-3 SN number and diameter, and EO SN number were 2.0 ± 1.0, 3.9 ± 1.2, 0.2 ± 0.1 and 20.9 ± 22.1, respectively (mean ± standard error of the mean, s.e.m.). However, because the F_2 _individuals from this cross did not recapitulate the entire distribution of cavefish VAB phenotypes (Figure 1A), we crossed another pair of F_1 _progeny from the same P_0 _to generate an additional F_2 _family. We used marker-assisted selection to choose three pairs of F_2 _individuals from this family that were hetero- or homozygous for cavefish alleles at two putative VAB and three SO-3 SN QTL loci (see Methods). We then crossed these F_2 _in order to generate 91 F_3 _individuals that recovered the entire cavefish VAB phenotype, although the distributions for SO-3 and EO SN remain somewhat restricted (Figure 1A-D). In combination with the original 384 F_2_, these additional F_3 _were included to increase our ability to detect correlations and QTL for the VAB, SN and eye size phenotypes.

### VAB and eye size are strongly correlated with EO SN but not SO-3 SN

Since SN are the sensory receptors that facilitate VAB [27], we expected the number of SN to be strongly correlated with the level of VAB in our hybrid families. In a previous study using cavefish, surface fish and their F_1 _progeny, we found a significant positive correlation between VAB and the number of SN at SO-3, which are located in the cranial region immediately ventral to the EO [27]. However, in the present study using both F_2 _and F_3 _hybrids, we found that the correlation between these two traits was not significant (*r *= 0.13, *P *= 0.052; Figure 1G), although the correlation between VAB and SN diameter at SO-3 was significant (*r *= 0.14, *P *= 0.044; Figure 1H). This new result suggests that the number of SN at SO-3 may not be the most important parameter determining VAB. After further examination of SN within the cranial regions of surface fish and cavefish, we found that SN are located in the EO in cavefish (yellow dotted line in the bottom of Figure 1N) and in F_2 _and F_3 _hybrids with reduced eyes, but not in surface fish (yellow arrow in the bottom of Figure 1L, M: F_3_; Figure 1K: surface fish). In contrast to the results for SO-3 SN, we found that the number of EO SN was positively correlated with VAB (*r *= 0.29, *P *< 0.001; Figure 1F) and negatively correlated with eye size (*r = *-0.44, *P *< 0.001; Figure 1J), and that eye size was also negatively correlated with VAB (*r *= -0.26, *P *< 0.001; Figure 1I). These results suggest that VAB may be mediated by an increase in EO SN and that both traits are negatively correlated with eye size.

### Genetic linkage mapping and QTL analysis

**Figure 2:**
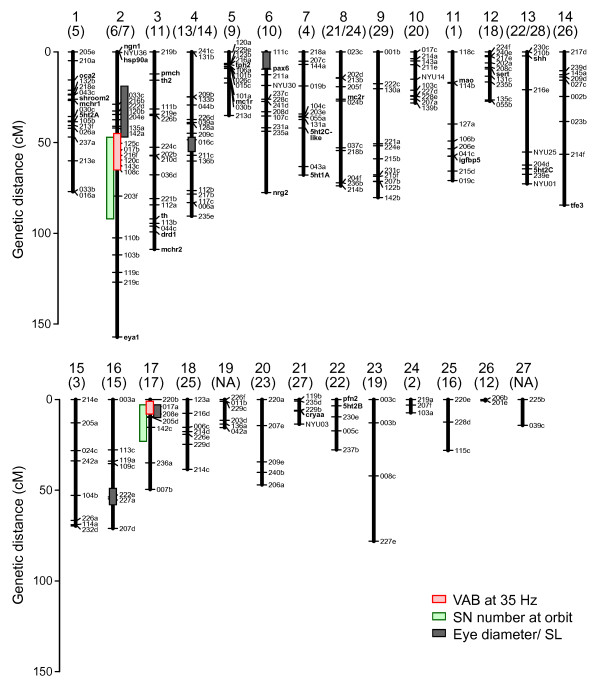
***Astyanax mexicanus *genetic linkage map from a Texas surface fish × Pachón cavefish cross**. The names of the genomic markers are indicated at the right of each linkage group. LG ids for this cross are shown at the top with LG ids corresponding to the numbering scheme of Protas *et al. *[23] made from the F2 progeny of Mexican surface fish × Pachón cavefish cross shown in parentheses. NA indicates that there is no homologous linkage group in Protas *et al. *[23]. The bars denote Bayesian credible intervals with probability coverage as 0.95. New genes placed on the map in this study were the 5-hydroxytryptamine (serotonin) receptors (*5ht1A, 5ht2A, 5ht2B, 5ht2C, 5ht2C_like*), dopamine receptor D1 (*drd1*), eyes absent homolog 1 (*eya1*), monoamine oxidase (*mao*); melanin-concentrating hormone receptor 2 (*mchr2*), neurogenin 1 (*ngn1*), neuregulin 2 (*nrg2*); pro-melanin concentrating hormone (*pmch*); profilin 2 (*pfn2*); serotonin transporter (*sert*); tyrosine hydroxylase 1 and 2 (*th, th2*); and tryptophan hydroxylase 2 (*tph2*). Other genes (*cryaa, hsp90a, igfbp5, mc1r, mc2r, mch1r, oca2, pax6, shhA, shroom2, tfe3*) were genotyped and mapped as in previous studies [7,22,34].

To determine the number and position of the genetic loci controlling VAB, SN number and eye size, we first constructed a genetic linkage map of the *Astyanax *genome from 246 markers genotyped among 384 F_2 _individuals. Most of the markers represent randomly-distributed microsatellite polymorphisms, but we also included 28 markers representing candidate genes for SN development, behavioral variation and other previously mapped traits (for example, *ngn1*, *eya1*, *nrg2*, the *5ht *serotonin receptors, the *mao *serotonin enzyme, the *sert *serotonin transporter, *shh*, *pax6 *and *oca2*) [7,23,42]. Our linkage map recovered 27 linkage groups (LG) spanning 1,513 cM (Figure 2). Since *Astyanax *have 25 haploid chromosomes [43], this map likely covers the entire genome. Our map is comparable to a previously published *Astyanax *genetic map [23] constructed from a cross between a Mexican surface fish and Pachón cavefish. Small discrepancies in marker order and inter-marker distance between this previous map and ours are likely due to the use of different surface fish populations.

Using this linkage map, we performed three types of QTL scans: (1) classic interval mapping with a single-QTL model treating the F_2 _and F_3 _separately, as well as combining these F_2 _and F_3 _datasets; (2) a modified single-QTL analysis using both F_2 _and F_3 _but accounting for kinship among these generations in QTLRel [44]; and (3) multiple QTL mapping with both F_2 _and F_3 _as implemented by the function *stepwiseqtl *in R/qtl [45,46]. By comparing the results of the first and second analyses (Additional file 1, the left and right column), we assessed the consistency of our QTL scans as well as the potential noise caused by using multiple generations. If the results were similar and relatively unaffected by kinship (see variance components in Additional file 2), we then applied the multiple QTL scan in *stepwiseqtl *since our Castle-Wright estimates suggest that a minimum of two or more loci control most traits (see above). We analyzed six traits using the classic and modified single-QTL models (VAB, SO-3 SN number, SO-3 SN diameter, EO SN number, eye size/SL and, as a control, albinism). We found similar results for all traits except SO-3 SN number and diameter (Additional file 1D, E). These two traits exhibited large variance components due to kinship and we thus dropped them from further analysis. The variance components of the remaining traits were small (Additional file 2). Using *stepwiseqtl *with both F_2 _and F_3 _to analyze albinism and eye size, we were able to detect two previously-reported QTL for albinism at the *oca2 *gene locus on LG1 [23] (Table 1, Additional file 1F, G) and for eye size on LG 2 [33]. We conclude that we can safely drop relatedness for the remaining traits and, therefore, show the results for multiple QTL mapping using the combined F_2 _and F_3 _datasets.

**Table 1:** A summary of the location and effect size of significant QTLs for **six **traits

Trait	N	Linkage Group	Position	LOD	PVE	Effect size and direction
**VAB**	227				Total: 19.79	**√NOA **(0 to 7.68)
		2	51.0	3.85	6.52	+1.11
		17	4.0	5.69	9.81	+1.22
**EO SN number**	253				Total: 18.54	**SN number **(0 to 10)
		2	75.0	5.74	8.98	+2.02
		17	8.0	3.84	5.89	+1.51
**Eye diameter/SL**	350				Total: 40.13	**Eye-size/SL **(0.0558 to 0.1382)
		2	28.9	10.22	8.62	-0.0128
		4	48.4	4.93	4.01	+0.0068
		6	4.0	5.44	4.44	-0.0065
		16	54.0	7.83	6.50	-0.0078
		17	8.0	9.46	7.94	-0.0111
**Albinism**	254					**Albino **(0) - **Pigmented **(1)
		1	16.0	55.91	63.71	-0.86

N is the number of F_2 _and F_3 _individuals used, LOD is the log of odds ratio, and PVE is the percentage of phenotypic variance explained. The summed PVE of all QTL are shown at the top row of each trait (at Total:). Effect size and direction (positive and negative) of QTL refers to the magnitude of shift between homozygotes of surface fish alleles and cavefish alleles. The numbers in parentheses indicate the ranges of phenotypes. Three QTL for eye diameter/SL at LG 4, 6 and 16 require further analyses to be confirmed (see Results).

### Two QTL for cavefish VAB at 35 Hz but not other frequencies

**Figure 3:**
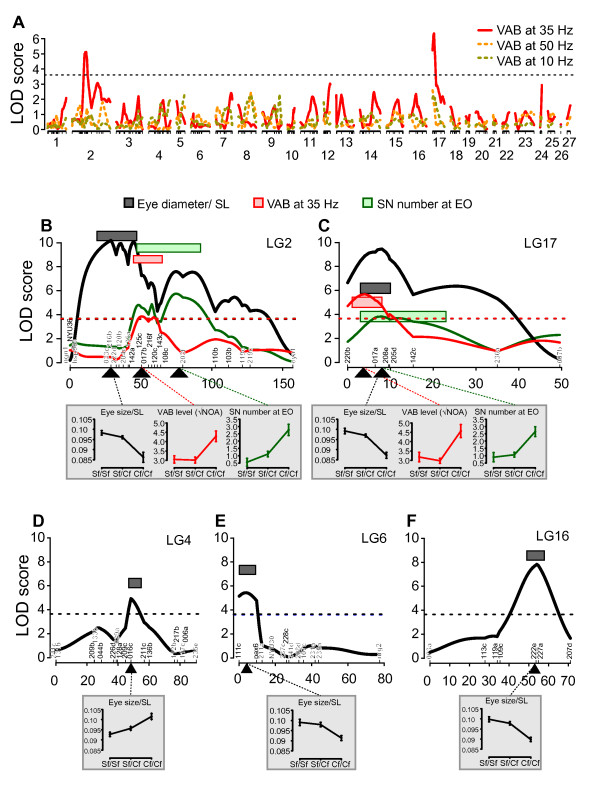
**QTL mapping of VAB, SN number and size, and eye size**. (**A**) LOD scores computed with single-QTL model genome-scan are plotted against the distance across each linkage group (LG). Red solid lines indicate LOD scores for VAB-level at 35 Hz vibration stimulus, yellow dotted lines for VAB at 50 Hz stimulus, and lime green dotted lines for VAB at 10 Hz stimulus. Significant VAB QTL were detected at LG2 and 17 only at 35 Hz stimulus. The horizontal line indicates the genome-wide significance thresholds at *P *< 0.05. (**B, C**) LOD scores computed following multiple QTL mapping. Nine QTL were found across five different LG (see also Table 1). Six overlapping Bayesian credible intervals of QTL for VAB, EO SN number and eye size were found on LG 2 and 17. The X-axis indicates genetic distance in centimorgans (cM), and colored bars denote Bayesian credible intervals with probability coverage as 0.95 for each significant QTL. Insets with gray background show effect plots of phenotypic values against each genotype (mean ± s.e.m.) at the peak locus denoted by the black triangle. Sf/Sf, surface fish homozygote, Sf/Cf, heterozygote, and Cf/Cf, cavefish homozygote. Horizontal dotted lines are genome-wide significant thresholds (*P *< 0.05) calculated from the single-QTL model (see Methods).

We initially surveyed QTL for VAB at different vibration stimuli. As reported previously, cavefish show the strongest VAB at 35 Hz and intermediate-levels at 10 and 50 Hz; additionally, some surface fish show an intermediate-level of VAB at a broad range between 5 and 35 Hz [27,40]. By performing an abbreviated QTL scan using the single-QTL model, we detected two significant QTL for VAB, but only with the 35 Hz stimulus (Figure 3A). This indicates that cavefish VAB at 35 Hz has a genetic basis. The same VAB QTL were revealed using *stepwiseqtl *mapping below.

### Two QTL clusters control VAB, EO SN number and eye size

By performing *stepwiseqtl*, we detected nine QTL: two each for VAB and EO SN number on LG 2 and 17, and five for eye size on LGs 2, 4, 6, 16, and 17 (Figure 3, Table 1). The QTL for VAB and EO SN number were relatively consistent across all types of analyses, allowing for some variation since single-QTL models are not designed to detect QTL for complex traits. The eye size QTL on LG 2 and 17 were also consistent among analyses, but the eye QTL on LG 4, 6 and 16 were not evident in the single-QTL scans (Additional file 1B, left column). Although this result may be expected since eye size is genetically complex in *Astyanax *[33], these three QTL will need to be confirmed in future analyses. We did not detect significant epistasis among any of these QTL (Figure 3, Table 1). For each phenotype, the sum of detected QTL accounted for 19.8%, 18.5% and 40.1% of the total phenotypic variance in VAB, EO SN number and eye size, respectively (Table 1). Although the QTL for these three traits were found on five different linkage groups distributed throughout the *Astyanax *genome, the Bayesian credible intervals for six VAB, EO SN and eye size QTL formed congruent or overlapping clusters on LGs 2 and 17 (1 cM overlap at the 47 cM position in LG 2, and 5 cM overlap at the 3 to 8 cM position of LG 17; Figures 2 and 3B, C). Particularly noteworthy, the credible intervals for the two most significant eye size QTL overlap with the two QTL for VAB and EO SN number (Figure 3B, C). The observed number of QTL clusters for these three traits is significantly higher than expected by under a Poisson distribution (*Χ*^2 ^= 98.2, *df *= 3, *P *= 3.8 × 10^-21^) [47]. Since alleles located within short genomic regions are generally inherited together as a unit, these QTL clusters support the physical correlations we found for VAB, EO SN number and eye size (Figure 1F, I, J).

Further examination of these overlapping QTL supports the hypothesis that VAB is enhanced by expanding the number of SN within the EO at the cost of eyes. We found that the cavefish alleles at each QTL shift the VAB, SN and eye size phenotypes in the direction expected given the distribution of these phenotypes between the surface fish and cavefish P_0_; when VAB level was high there were more SN and smaller eyes than when VAB level was low or absent (Table 1; see insets of Figure 3B, C; and Additional file 3). None of the 28 candidate genes were associated with any of the 9 QTL. As reported previously [7,33], the *shhA *locus was not located in or near any of the eye size QTL (see at LG13 of Figure 2). In addition, *alphaA crsytallin *(*cryaa*) and *shroom2*, which were located within eye QTL in a previous study [3], did not map to an eye size QTL in the present analysis. The significant clustering of VAB, EO SN and eye size QTL on LG 2 and 17 supports the conclusion that the genetic factors responsible for VAB and EO SN enhancement are also responsible for eye regression, either as a result of genetic hitchhiking or pleiotropy.

### The EO SN are major receptors for VAB

**Figure 4:**
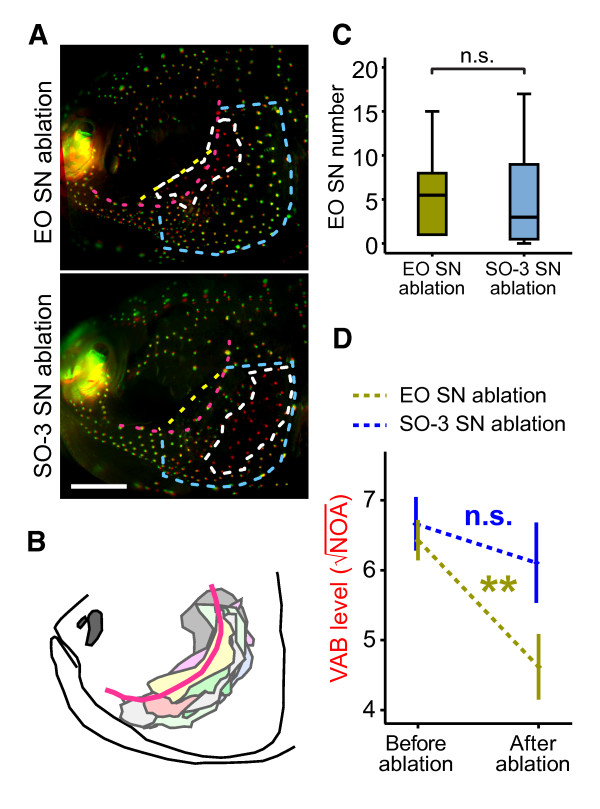
**Effects of bilateral SN ablation on VAB in cavefish**. (**A**) Comparison of DASPEI-stained EO SN and SO-3 SN in the ablated areas. The SN present before but absent after ablation within the two ablated regions (outlined by dashed white lines) are pseudo-colored in red. The red dashes indicate the line of suborbital canal neuromasts in the head lateral line and the areas enclosed by the blue dashed lines indicate the SO-3 region; the areas between the suborbital canal lateral line (red dashes) and dashed yellow lines represent the region containing EO SN. Scale bar in (lower panel of A) is 1.0 mm. (**B**) Schematic drawing of the ablated areas of 10 cavefish in the EO SN ablation experiment. Each experiment was color-coded. The snout is shown at the left. The ablated area included both the EO region and a part of the SO-3 region. The suborbital canal lateral line is indicated with red line. (**C**) The EO SN number of cavefish prior to ablation of EO or SO-3 SN. There was no difference in EO SN number between these two groups (*Z *= -0.54, *P *= 0.614; n = 10 for EO SN ablation; n = 7 for SO-3 SN ablation). (**D**) VAB in cavefish before SN ablation and four to six days after SN ablation. Values are means ± s.e.m. **: *P *< 0.01. n.s.: not significant. Number of cavefish used were: n = 10 for EO, n = 7 for SO-3. Rod vibration at 35 Hz was used to measure VAB.

The results of the quantitative genetic analyses suggest that VAB and EO SN number are phenotypically and genetically linked to eye size in *Astyanax *cavefish. These results raise the intriguing possibility that VAB is facilitated by an increase in EO SN in cavefish. To test this hypothesis, we compared the level of VAB among cavefish whose area of EO or SO-3 SN were ablated. Following EO SN ablation, cavefish showed a significant decrease in VAB (paired *t-*test: *t_9 _*= 3.66; *P *= 0.005; Figure 4A, D; ablated areas in the EO region are shown in Figure 4B; EO SN numbers before ablation are shown in Figure 4C). In contrast, cavefish showed only a minor reduction in VAB following ablation of SO-3 SN, which was not significant (paired *t-*test: *t_6 _*= 0.68; *P *= 0.524; Figure 4D). Although the EO SN ablation included a part of the SO-3 region, as well as some canal neuromasts (Figure 4B), EO SN were consistently removed, whereas SO-3 ablation removed more SN than the total number of EO SN (number of ablated SN in the EO SN ablation: 38.5 ± 7.8; in the SO-3 SN ablation: 98.0 ± 27.1; mean ± s.e.m.; *t*_15 _= -2.45, *P *= 0.027). Thus, even though cavefish have fewer EO SN than SO-3 SN, these ablation experiments provide strong evidence that SN in the EO area are the main sensory receptors responsible for VAB.

### shh-mediated eye degeneration is insufficient to facilitate VAB and EO SN

**Figure 5:**
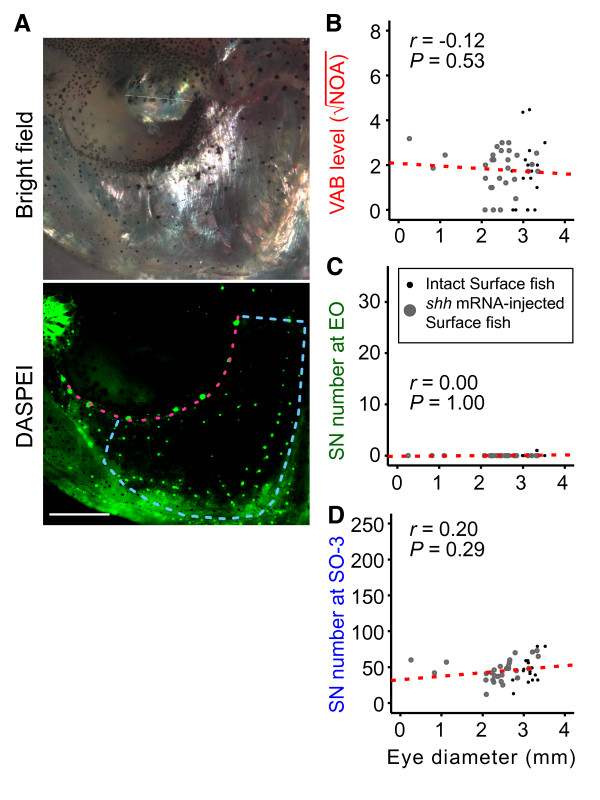
**Effects of *shh *induced eye degeneration on VAB and SN number**. (**A**) Bright field image (upper) and DASPEI-stained neuromasts (lower) in two-year-old adults that developed from *shhA*-mRNA injected surface fish embryos. The red dashes indicate the line of suborbital canal neuromasts in the head lateral line and the areas enclosed by the blue dashed lines indicate the SO-3 region. Scale bar in (lower panel of A) is 1.0 mm. (**B**-**D**) Regression analysis showing the relationship between eye diameter and (B) VAB level, (C) EO SN number and (D) SO-3 SN number in *shhA*-mRNA injected surface fish (gray dots) compared to uninjected surface fish (black dots). Linear regression line is shown in red. Eye degeneration did not result in the appearance of SN in the EO or increase VAB level in *shh*-overexpressed surface fish.

**Figure 6:**
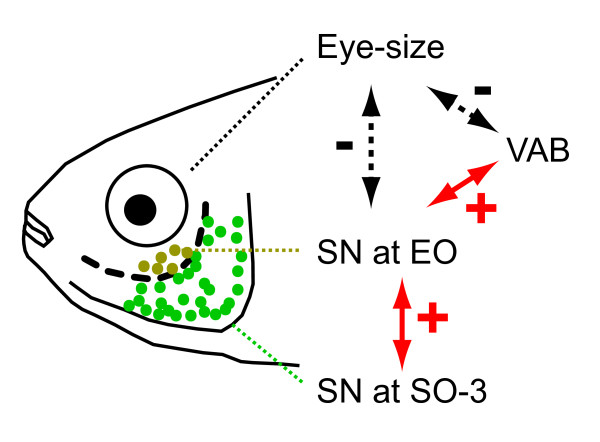
**Schematic diagram showing correlations among four traits examined in this study**. Yellow and green dots represent EO SN and SO-3 SN, respectively. Red arrows indicate significant positive correlations (denoted by "+"), shown in this study and previous studies [27]. The statistics of positive correlation between EO SN and SO-3 SN were *r *= 0.24, *P *< 0.001, *n *= 247. Black arrows with dotted lines indicate significant negative correlations (denoted by "-") determined in the present study.

Although the genes that increase VAB and EO SN may drive eye regression through genetic hitchhiking or pleiotropy (see also Figure 1I, J), it is still possible that reduced eye size alone may increase VAB. To test this alternative possibility, we further compared the level of VAB among surface fish with experimentally regressed eyes following *shhA *overexpression [26]. In addition to eye size, expanded *shh *expression is also known to increase jaw width and taste bud number in cavefish [11], suggesting that this pathway could potentially alter SN number or VAB. Following *shhA *mRNA overexpression, eye diameter varied considerably among surface fish (horizontal axis on Figure 5A-D), but was not associated with a significant increase in VAB (*r = *-0.12, *P = *0.53), EO SN (*r *= 0.00, *P *= 1.00), or SO-3 SN (*r *= 0.20, *P *= 0.29) (Figure 5B-D). These results suggest that *shh*-induced eye regression is insufficient to increase EO SN and VAB and that the molecular pathway decreasing eye size concomitantly with VAB and EO SN enhancement is parallel to the SHH pathway. We conclude that the same or closely linked genetic factors responsible for the enhancement of EO SN and VAB are also responsible for the reduction in eye size (Figure 6).

## Discussion

We have shown that eye regression in *Astyanax *cavefish is phenotypically and genetically correlated with the evolution of VAB, an adaptive trait controlling feeding efficiency [27], as well as the enhancement of its sensory receptors, SN specifically localized within the EO. Genetic mapping studies revealed clusters of congruent or overlapping QTL on LG 2 and 17 that control eye size, VAB and the number of EO SN. In addition, we have also demonstrated a significant role of the EO SN in VAB, and that the open space created by *shh*-induced eye degeneration is insufficient to promote VAB or SN enhancement. This experimental result further highlights the importance of the genetic linkage between eye loss and the VAB sensory system. Our results support the hypothesis that selection for an adaptive behavior and its underlying sensory receptors are enhanced in cavefish at the expense of eyes. To our knowledge, this is the first study to report phenotypic and genetic relationships between a regressive phenotype, an adaptive behavior and the sensory system responsible for this behavior.

We have discovered a significant genetic correlation between VAB and EO SN number but not between VAB and SO-3 SN number and size. This result was surprising in light of our previous studies, which found that removal of SO-3 SN reduced (but did not eliminate) VAB [27]. A possible explanation for this discrepancy is that the physical SN ablation method used in our previous study did not discriminate effectively between EO SN and SO-3 SN, which are present in adjacent regions in the cavefish cranium, or that the actual contribution of SO-3 SN to VAB is minor compared to that of EO SN. Our current ablation study supports either explanation by showing that EO SN are the major VAB receptors, whereas SO-3 SN, despite their larger number, have only a minor role in VAB (Figures 1 and 4). Although our EO SN ablation experiment also ablated some SO-3 SN and canal neuromasts, we conclude that the latter receptors play a minor role in VAB for two reasons. First, in the SO-3 SN ablation experiment, more SN were ablated than EO SN. Yet, despite the loss of more SN in SO-3, the level of VAB was not attenuated significantly (Figure 4). In contrast, deleting a much smaller population of EO SN resulted in a dramatic and statistically significant reduction in VAB (Figure 4). Second, in several former studies, the involvement of canal neuromasts in VAB was excluded through three observations: (1) selective SN ablation was enough to decrease VAB even while maintaining canal neuromasts [27], (2) cavefish VAB was tuned to the frequency at the peak sensitivity of SN but not of canal neuromasts [27], and (3) the selective interference with canal neuromasts failed to attenuate the response [37]. So what makes EO SN necessary for VAB? It is possible that there are structural or neural circuitry differences between the two populations of EO and SO-3 SN that may account for our results, or that the EO SN are better positioned above the eye socket to detect fluctuations in water due to the bulging shape of the adipose tissue plug that fills the eye orbits of cavefish [5] or bulging eye balls of F_2 _and F_3 _hybrids [48]. However, since the level of VAB did not drop to zero in our EO SN ablation, it seems likely that other SN contribute to VAB as well, possibly including SN at SO-3 and on the dorsal trunk [27].

Our present investigation has also clarified the difference between the strong type of VAB observed in cavefish (10+ approaches towards a vibrating object) and the weaker form previously detected in a small proportion of surface fish raised in the laboratory (> 4 approaches) [27,40]. Cavefish VAB peaks at 35 Hz, whereas surface fish VAB shows a continuous low activity from 5 to 35 Hz [27,40]. In this study, VAB showed two genetic components detectable at 35 Hz, but none at 10 or 50 Hz, suggesting that the weak form of surface fish VAB appears without a major genomic change and could be accomplished through environmental effects and/or developmental plasticity. Considering that at least three cavefish populations (Pachón, Los Sabinos and Piedras cavefish) exhibit a strong form of VAB [27,49], VAB may, therefore, represent a broadly distributed evolutionary trait in cavefish. The elaboration of SN at a new site, the EO, could have been a key factor in the evolution of VAB during adaptation to subterranean habitats [10,40]. Determining the mechanisms underlying EO SN development and VAB among independently evolved cave populations [50,51] could open a path to understanding the origins of neurological and behavioral novelty during evolution.

The major conclusions of this study are that EO SN number is strongly correlated with both VAB and eye size, and that all three traits are controlled by QTL on LGs 2 and 17 with congruent or overlapping Bayesian credible intervals. These results suggest that the gene loci responsible for VAB, EO SN number and eye-size may be subject to possible physical linkage and genetic hitchhiking or may in fact be the same genes with pleiotropic effects. The existence of QTL clusters could facilitate rapid adaptation to environmental changes by concurrently fixing multiple beneficial traits. Previous QTL analyses have also shown tight linkages and overlapping QTL for several other cavefish traits, including eye size, lens size, taste bud number, tooth number, melanophore number and chemical sense ability [7,33]. This observation suggests that adaptation to cave life in *Astyanax *is the result of tradeoffs among many phenotypic traits, possibly through functional shifts within pluripotent neural crest cells which contribute to most of the above traits through developmental processes [52]. However, one must note that widespread clustering of QTL does not necessarily entail a phenotypic tradeoff. In a previous scan of QTL in *Astyanax*, Protas *et al. *reported the overlap of six QTL for eye size and melanophore number (MelE), yet the phenotypic correlation between these traits is only *r *= -0.05 [7,33].

The present investigation provides a clear example of a tradeoff between constructive traits (VAB and EO SN) and a regressive trait (eye size) in parallel to the SHH-signaling pathway. The short genomic distance between the QTL for eye regression, EO SN and VAB [11] could be a key factor in promoting the rapid adaptation of *Astyanax *to a novel environment. In other rapidly evolving animals, such as African cichlids and three-spined sticklebacks, QTL clusters have also been shown to control groups of potentially advantageous traits, including sex determination and body color, and body shape and plate number [53,54].

## Conclusions

We have discovered a significant genetic correlation among eye size, VAB and EO SN number. Our results, therefore, support the hypothesis that eye loss has evolved in *Astyanax *cavefish as a result of indirect selection against eyes due to a trade-off between two constructive traits, VAB and EO SN enhancement, based either on genetic hitchhiking or pleiotropy. There has been a long-standing debate on the neutrality or benefit of eye regression in cave animals [1,2,4,34,35]. Since cave-adapted arthropods exhibit reduced eyes and elongated sensory antennae [1,2,8], the loss of eyes as a result of selection for increased tactile sensitivity is an attractive general hypothesis for the convergent evolution of eye regression in cave fauna.

## Methods

### Biological materials and crosses

*Astyanax mexicanus *surface fish used in this study were laboratory raised descendants of original collections made in Balmorhea Springs State Park, Texas, and cavefish were laboratory raised descendants of original collections from Cueva de El Pachón (Pachón cavefish) in Tamaulipas, Mexico. We generated all hybrid progeny from an original mating between one pair of cavefish and surface fish [11,41,55,56]. One pair of F_1 _hybrids from this cross was mated to generate 384 F_2 _individuals for phenotypic and genetic analysis, while another pair of F_1 _hybrids was crossed to generate a second group of about 200 F_2 _progeny. These F1 hybrids were randomly selected. We genotyped this second set of hybrids at markers 017a, 215d, 145a, 204d and 218e, which are near potential VAB (017a and 215d) and SN (145a, 204d and 218e) loci identified by a preliminary QTL scan using 384 F_2 _individuals. To overcome a reduced rate of spawning in this group, we chose three pairs of F_2 _progeny that were either homozygous or heterozygous for the cavefish alleles at the marker loci and crossed them to generate 91 F_3 _progeny. Although these hybrids exhibited a reduced rate of spawning, there was no noticeable difference in their survivability. Hybrids were fed living *Artemia *larvae and were maintained individually in 500 ml tanks or in groups of approximately 10 in 3 L tanks. We phenotyped each hybrid once for SN number and eye size and two or three times for VAB when they were one to two years old. Because of the limitations of tank size [57], hybrids of different ages approximate the same standard length (approximately 3 cm) and there is no positive relationship between age and SN number at either SO-3 or the EO (Additional file 4). Additionally, the results of our QTL scans did not differ when including age as a covariate (data not shown). The animal procedures used in this study were approved by the University of Maryland Animal Care and Use Committee and conform to NIH guidelines.

### Marker genotyping

We isolated genomic DNA from fin-clips using the DNeasy Blood & Tissue Kit (Qiagen, Valencia, CA, USA) or the quick extraction protocol [58]. We genotyped fish for 226 genome-wide polymorphic microsatellite loci described previously [23] and for polymorphisms within or near 20 candidate genes for SN number and behavioral variation. We successfully genotyped 93,208 (95.6%) of these microsatellite markers among 382 F_2 _and 11,375 (95.5%) of 125 selected microsatellite markers among 91 F_3_. Candidate gene homologues were identified in the fugu, medaka, stickleback, tetraodon and zebrafish genomes. Degenerate primers were designed to match the most conserved amino acid regions using CODEHOP [59] or SCPrimer [60]. We performed degenerate PCR on genomic DNA extracted from the fin-clips of P_0 _surface fish and Pachón cavefish using Takara LA Taq DNA polymerase (Clontech, Mountain View, CA, USA) and the following touch-down program: 1 cycle at 94°C for 90 sec; 3 cycles each at 94°C for 20 sec, 63°C for 20 sec and 68°C for 2.5 minutes; 3 cycles each at 94°C for 20 sec, 61°C for 20 sec and 68°C for 2.5 minutes; 3 cycles each at 94°C for 20 sec, 59°C for 20 sec and 68°C for 2.5 minutes; 30 cycles at 94°C for 20 sec, 57°C for 20 sec and 68°C for 2.5 minutes; and finally 1 cycle at 72°C for 4 minutes, followed by a 4°C incubation. PCR fragments were characterized by either direct sequencing with the same degenerate primer used for PCR or by conventional sequencing after sub-cloning into the pCRII plasmid using the TOPO TA Cloning Kit (Invitrogen, Grand Island, NY, USA). The homology of each candidate gene fragment was confirmed by blastn. After sequencing each candidate gene region in surface fish and Pachón cavefish, the sequences were extended with primers designed from known sequences using the GenomeWalker™ Universal Kit (Clontech), and aligned using either Vector NTI (version 7, InforMax, Bethesda, MD, USA) or Sequencher™ (version 5, Gene Codes Corp., Ann Arbor, MI, USA) software to identify microsatellite and single nucleotide polymorphisms (SNPs). Microsatellite polymorphisms were genotyped as described by Protas *et al. *[23] after designing primers in Primer3Plus [61]. SNPs were genotyped by either Taqman^® ^(Roche Applied Science, Indianapolis, IN, USA) or HybProbe (Roche Applied Science) technology. Primers and fluorescent probes were designed using Primer Express (version 3.0, Applied Biosystems, Carlsbad, CA, USA) or LightCycler^® ^Probe Design Software 2.0 (Roche Applied Science), respectively. The primer and probe sequences for these candidate genes are provided in Additional file 5.

### Vibration attraction behavior

We assayed VAB as described previously [27,49]. Briefly, four or five days before the beginning of an assay, individuals were acclimated in a cylindrical assay chamber (Pyrex 325 ml glass dish, 10 cm diameter × 5 cm high, Corning, Corning, NY, USA) filled with conditioned water (pH 6.8; conductivity approximately 600 μS). During the assays, vibration stimuli were created using a 7.5 mm-diameter glass rod vibrating at 35 Hz using a Leader LG1301 function generator (Leader Instruments Corp., Cypress, CA, USA) with an audio speaker (Pro Speakers, Apple, Cupertino, CA, USA). The number of approaches (NOA) to the vibrating rod was video recorded during a three-minute period under infrared illumination (880 nm wave length, BL41192-880 black light, Advanced Illumination, Rochester, VT, USA), and counted using ImageJ 1.42q software (NIH, Bethesda, MD, USA).

### Eye measurements

Eye size was determined from photographs of each fish by digitally measuring the diameter of each eyeball along its rostral-caudal axis using ImageJ. We standardized these measurements by dividing them by each fish's standard length (SL), the length of the body from the tip of the snout to the base of the caudal fin.

### Neuromast vital staining

Neuromasts were vital stained as described previously [27,49]. Briefly, fish were immersed in 25 μg/ml 2-(4-(dimethylamino)styryl)-N-ethylpyridinium iodide (DASPEI; Invitrogen, Eugene, OR, USA) [62] dissolved in conditioned water for one hour, followed by immersion in ice-cold 66.7 μg/ml Ethyl 3-aminobenzoate methanesulfonate salt (MS222, Sigma) in conditioned water. The specimens were viewed under a fluorescence microscope (Axioskop 2 equipped with 2.5× Plan-Neofluar lens with a numerical aperture of 0.075 and a FITC filter set; Zeiss, Göttingen, Germany); we then photographed the fish with a Zeiss Axiocam CCD camera. Neuromasts were quantified on images of DASPEI-stained fish using ImageJ software. SN were counted both in the epidermis over the cranial third suborbital (SO-3) bone [41] and within the orbit epidermis dorsal to the line of the suborbital canal neuromasts (indicated by red dashed line in Figure 1L-N). To determine SN size, the long diameter of the 10 largest SN in the same area was measured and averaged.

### Linkage and QTL mapping

Following marker genotyping, we used MapManager QTXb20 [63] software to construct an initial linkage map of the SNP and microsatellite markers using 384 F_2 _fish with complete genotype data and the Kosambi map function. We then used the program R/qtl [45] to identify statistically unlikely events, such as double crossovers in adjacent intervals or single crossovers in a small interval under maximum likelihood estimation [64], to refine marker order and to estimate inter-marker distances.

We performed QTL mapping using two strategies: (1) multiple interval mapping via the function *stepwiseqtl *in R/qtl [45]; and (2) single-QTL model mapping accounting for relatedness in the program QTLRel [44]. All logarithms of the odds (LOD) significant thresholds were determined following 2,000 permutation tests after single-QTL model mapping with *scanone *implemented in R/qtl or *scanOne *in QTLRel. The 95% Bayesian credible intervals for QTL positions, which are analogous to 95% confidence intervals, were obtained using R/qtl. For multiple QTL mapping, we first calculated genotype probabilities for any missing genotype using the *calc.genoprob *function before performing the forward/backward model selection function implemented in *stepwiseqtl*. Following model selection, we chose the model with the maximum penalized LOD score generated from 1,200 permutation tests [65]. For QTLRel mapping, we first generated pedigree charts for the F_2 _and F_3 _hybrid families and then calculated condensed identity coefficients and estimated variance components with the *cic *function. We used these variance components when scanning the genome for QTL using the *scanOne *function (see Additional file 2). The genome-wide significant thresholds (*P *< 0.05) for this latter analysis were calculated from 2,000 permutation tests.

### SN microablation

SN were ablated by a modification of our previous method [27]. Prior to ablation, we measured VAB and counted neuromasts stained with DASPEI. Only cavefish showing a high VAB level (more than 16 NOA) were selected for SN ablation. SN were ablated by applying Vetbond non-toxic tissue adhesive (3M, St. Paul, MN, USA) either to the EO or the SO-3 region using a micro nylon loop, a traditional embryological tool [58]. After application of tissue adhesive to one side, cavefish were exposed to air for 10 seconds, and tissue adhesive was then applied to the same area on the opposite side, followed by a second cycle of air-drying. The treated fish were placed in a 10 cm-diameter cylindrical chamber containing conditioned water at room temperature. Within a day of active swimming the tissue adhesive usually peeled off the body, resulting in a void in the underlying field of SN. After four to six days of acclimation/recovery, SN ablated fish were subjected to the VAB assay followed immediately by staining for one hour with 25 μg/ml DASPEI.

### shh overexpression

Overexpression was carried out by injecting zebrafish *shhA *mRNA (20 to 80 pg) into two to four cell stage embryos as described previously [26]. Capped *shhA *mRNA was synthesized from the pSP64T plasmid using the mMESSAGE mMACHINE^® ^SP6 Kit (Life Technologies, Grand Island, NY, USA).

### Statistics

Correlation studies were conducted using IBM SPSS 20.0.0 software (IBM, Somers, NY, USA). Castle-Wright estimates were performed according to Lynch and Walsh [66]. Significance of QTL clustering was performed using a goodness-of-fit [47]. We divided the 1,500 cM *Astyanax *genome into 50 bins of 30 cM each and counted the number of bins with 0, 1, 2 and 3 QTL. We then compared this observed distribution to that expected from a Poisson distribution where the average number of QTL expected per bin is 0.18 (9 QTL divided by 50 bins of 30 cM each).

## Abbreviations

DASPEI: 2-(4-(dimethylamino)styryl)-N-ethylpyridinium iodide; EO: eye orbit; LG: linkage groups; LOD: logarithms of the odds; MelE: melanophore number above the eye; NOA: number of approaches; QTL: quantitative trait locus; s.e.m.: standard error of the mean; *shh*: *sonic hedgehog*; SL: standard length of fish; SN: superficial neuromast; SNPs: single nucleotide polymorphisms; SO-3: suborbital bone 3; VAB: vibration attraction behavior.

## Competing interests

The authors declare that they have no competing interests.

## Authors' contributions

MY and WRJ conceived the project and designed the experiments. MY performed behavioral assays, vital dye labeling (DiI), morphometrics, cloning, most of the genotyping, QTL mapping, SN ablation studies, and a part of the *shh*-injection experiments and analyses, and generated the figures. YY performed some of the *shh*-injection experiments. KEO participated in the QTL mapping analyses. MY wrote the manuscript with significant contributions from KEO and WRJ. All authors read and approved the final manuscript.

## Supplementary Material

Additional file 1 **LOD scores from single-QTL mapping analyses with or without accounting for relatedness among the F_2 _and F_3_**. (**A-F**) LOD scores computed with classic single-QTL interval mapping using the F_2 _dataset (blue lines), F_3 _dataset (green lines), or F_2 _+ F_3 _combined dataset (red lines) in R/qtl (left column) and QTLRel (right column). In QTLRel, condense identity coefficients were calculated to adjust background variation according to kinship among the F_2 _and F_3 _generations. This analyses found (A) two QTL by the classic scan with the F_2 _and the F_3 _dataset (left column), or one QTL (right column) for VAB, (B) five QTL with the F_2 _and the F_3 _dataset (left at LG 2, 9, 17 and 23), or three QTL (right at LG2 and 17) for eye-size, (C) one QTL for EO SN number in both, no significant QTL for either (D) SO-3 SN number or (E) SO-3 SN diameter, and (F) one QTL for albinism in the both methods. (**G**) The LOD score from multiple QTL mapping of albinism also identified a single QTL at the *oca2 *locus, as described previously [23]. The horizontal lines indicate genome-wide significance of *P *< 0.05 based on 2,000 permutation tests. Click here for file

Additional file 2 **Variance Components calculated in QTLRel software**. The phenotypes of VAB, Eye size, EO SN number and albinism exhibited small variance components for both the additive and the dominance genetic matrixes, whereas SO-3 SN number and SO-3 SN diameter exhibited large variance components at the dominance genetic matrix. AA: additive genetic matrix; DD: dominance genetic matrix; and EE: the residual matrix. Click here for file

Additional file 3 **Scatterplots of phenotypic values against each genotype at the two cluster loci**. Scatterplots of phenotypic values of VAB (top row), eye size/SL (middle row) and EO SN number (bottom row) were shown at the marker position of 17b (left column, VAB and EO SN number), 222d (left column, eye size), and 17a (right column, all three traits) at linkage group 2 and 17. Blue dots and bars: F_2 _generation, Green dots and bars: F_3 _generation. Bars indicate mean ± s.e.m. Sf/Sf, surface fish homozygote; Sf/Cf, heterozygote; and Cf/Cf, cavefish homozygote. The cavefish alleles at each QTL cluster shift the distributions of VAB, SN and eye size phenotypes in the direction toward higher VAB level, more EO SN and smaller eyes. Click here for file

Additional file 4 **Phenotypic distributions between approximately one- and two-year-old fish used in current study**. The boxplot of phenotypic distributions were compared between approximately one- and two-year-old F_2 _fish. The increased VAB levels in two-year-old fish (top) was not associated with the increases of EO (middle; no significant difference: n.s.; The Mann-Whitney non-parametric test was applied since phenotypic values were not normally distributed), or SO-3 SN number (bottom; significantly decreased in two-year-old fish). N were indicated in the boxes. Click here for file

Additional file 5 **Candidate gene primer and probe sets used in this study**. Primers for the microsatellite markers were designed to amplify 150 to 350 base-pair genomic fragments containing length polymorphisms between surface fish and cavefish. Primers and fluorescent probes for Taqman and HybProbe genotyping methods were designed to detect single nucleotide polymorphisms between these two morphs. Click here for file

## References

[B1] Culver DC Cave Life, Evolution and Ecology 1982 Cambridge: Harvard University Press 189

[B2] Culver DC Pipan T The Biology of Caves and other Subterranean Habitats 2009 Oxford: Oxford University Press 254

[B3] Protas M Jeffery WR Evolution and development in cave animals: from fish to crustaceans Wiley Interdiscip Rev Dev Biol 2012 1 823 845 10.1002/wdev.61 PMC3620605 23580903

[B4] Darwin C On the Origin of Species Based on Natural Selection, or the Preservation of Favoured Races in the Struggle for Life 1859 6 London: John Murray 480

[B5] Wilkens H Evolution and genetics of epigean and cave *Astyanax-fasciatus *(Characidae, Pisces) - support for the neutral mutation theory Evol Biol 1988 23 271 367

[B6] Culver DC Wilkens H Wilkens H, Culver DC, Humphreys WF Critical review of the relevant theories of the evolution of subterranean animals Ecosystems of the World 2000 30 Amsterdam: Elsevier 381 398

[B7] Protas M Conrad M Gross JB Tabin C Borowsky R Regressive evolution in the Mexican cave tetra, *Astyanax mexicanus* Curr Biol 2007 17 452 454 10.1016/j.cub.2007.01.051 17306543 PMC2570642

[B8] Jernigan RW Culver DC Fong DW The dual role of selection and evolutionary history as reflected in genetic correlations Evolution 1994 48 587 596 10.2307/2410471 28568263

[B9] Schemmel C Vergleichende Untersuchungen an den Hautsinnesorganen ober- und unterirdisch lebender *Astyanax*-Formen Z Morph Tiere 1967 61 255 316 10.1007/BF00400988

[B10] Franz-Odendaal TA Hall BK Modularity and sense organs in the blind cavefish, *Astyanax mexicanus* Evol Dev 2006 8 94 100 10.1111/j.1525-142X.2006.05078.x 16409386

[B11] Yamamoto Y Byerly MS Jackman WR Jeffery WR Pleiotropic functions of embryonic sonic hedgehog expression link jaw and taste bud amplification with eye loss during cavefish evolution Dev Biol 2009 330 200 211 10.1016/j.ydbio.2009.03.003 19285488 PMC3592972

[B12] Jeffery WR Adaptive evolution of eye degeneration in the Mexican blind cavefish J Hered 2005 96 185 196 10.1093/jhered/esi028 15653557

[B13] Mitchell RW Russell WH Elliott WR Mexican Eyeless Characin Fishes, Genus Astyanax: Environment, Distribution, and Evolution 1977 89 Lubbock, Texas, USA: Texas Tech University Press

[B14] Jeffery WR Cavefish as a model system in evolutionary developmental biology Dev Biol 2001 231 1 12 10.1006/dbio.2000.0121 11180948

[B15] Jeffery WR Evolution and development in the cavefish *Astyanax* Curr Top Dev Biol 2009 86 191 221 19361694 10.1016/S0070-2153(09)01008-4 PMC3594791

[B16] Jeffery WR Emerging model systems in evo-devo: cavefish and microevolution of development Evol Dev 2008 10 265 272 10.1111/j.1525-142X.2008.00235.x 18460088 PMC3577347

[B17] Strecker U Hausdorf B Wilkens H Parallel speciation in *Astyanax *cave fish (Teleostei) in northern Mexico Mol Phylogenet Evol 2012 62 62 70 10.1016/j.ympev.2011.09.005 21963344

[B18] Ornelas-García CP Domínguez-Domínguez O Doadrio I Evolutionary history of the fish genus *Astyanax *Baird & Girard (1854) (Actinopterygii, Characidae) in Mesoamerica reveals multiple morphological homoplasies BMC Evol Biol 2008 8 340 10.1186/1471-2148-8-340 19102731 PMC2657800

[B19] Gross JB The complex origin of *Astyanax *cavefish BMC Evol Biol 2012 12 105 10.1186/1471-2148-12-105 22747496 PMC3464594

[B20] Bradic M Beerli P Garcia-de Leon FJ Esquivel-Bobadilla S Borowsky RL Gene flow and population structure in the Mexican blind cavefish complex (*Astyanax mexicanus*) BMC Evol Biol 2012 12 9 10.1186/1471-2148-12-9 22269119 PMC3282648

[B21] Gross JB Borowsky R Tabin CJ A novel role for Mc1r in the parallel evolution of depigmentation in independent populations of the cavefish *Astyanax mexicanus* PLoS Genet 2009 5 e1000326 10.1371/journal.pgen.1000326 19119422 PMC2603666

[B22] Menuet A Alunni A Joly JS Jeffery WR Rétaux S Expanded expression of sonic hedgehog in *Astyanax *cavefish: multiple consequences on forebrain development and evolution Development 2007 134 845 855 10.1242/dev.02780 17251267

[B23] Protas ME Hersey C Kochanek D Zhou Y Wilkens H Jeffery WR Zon LI Borowsky R Tabin CJ Genetic analysis of cavefish reveals molecular convergence in the evolution of albinism Nat Genet 2006 38 107 111 10.1038/ng1700 16341223

[B24] Varatharasan N Croll RP Franz-Odendaal T Taste bud development and patterning in sighted and blind morphs of *Astyanax mexicanus* Dev Dyn 2009 238 3056 3064 10.1002/dvdy.22144 19877280

[B25] Yamamoto Y Jeffery WR Central role for the lens in cave fish eye degeneration Science 2000 289 631 633 10.1126/science.289.5479.631 10915628

[B26] Yamamoto Y Stock DW Jeffery WR Hedgehog signalling controls eye degeneration in blind cavefish Nature 2004 431 844 847 10.1038/nature02864 15483612

[B27] Yoshizawa M Gorički Š Soares D Jeffery WR Evolution of a behavioral shift mediated by superficial neuromasts helps cavefish find food in darkness Curr Biol 2010 20 1631 1636 10.1016/j.cub.2010.07.017 20705469 PMC2946428

[B28] Duboué ER Keene AC Borowsky RL Evolutionary convergence on sleep loss in cavefish populations Curr Biol 2011 21 671 676 10.1016/j.cub.2011.03.020 21474315

[B29] Coombs S Patton P Windsor S Active wall following by Mexican blind cavefish (*Astyanax mexicanus*) J Comp Physiol A Neuroethol Sens Neural Behav Physiol 2010 196 853 867 10.1007/s00359-010-0567-8 20730435

[B30] Sharma S Coombs S Patton P De Perera TB The function of wall-following behaviors in the Mexican blind cavefish and a sighted relative, the Mexican tetra (*Astyanax*) J Comp Physiol A Neuroethol Sens Neural Behav Physiol 2009 195 225 240 10.1007/s00359-008-0400-9 19093125

[B31] Windsor SP Norris SE Cameron SM Mallinson GD Montgomery JC The flow fields involved in hydrodynamic imaging by blind Mexican cave fish (*Astyanax fasciatus*). Part II: gliding parallel to a wall J Exp Biol 2010 213 3832 3842 10.1242/jeb.040790 21037062

[B32] Elipot Y Hinaux H Callebert J Rétaux S Evolutionary shift from fighting to foraging in blind cavefish through changes in the serotonin network Curr Biol 2012 in press 10.1016/j.cub.2012.10.044 23159600

[B33] Protas M Tabansky I Conrad M Gross JB Vidal O Tabin CJ Borowsky R Multi-trait evolution in a cave fish, *Astyanax mexicanus* Evol Dev 2008 10 196 209 10.1111/j.1525-142X.2008.00227.x 18315813

[B34] Porter ML Crandall KA Lost along the way: the significance of evolution in reverse Trends Ecol Evol 2003 18 541 547 10.1016/S0169-5347(03)00244-1

[B35] Barr TC Cave ecology and the evolution of troglobites Evol Biol 1968 2 35 102

[B36] Parzefall J Field observation in epigean and cave populations of Mexican characid *Astyanax mexicanus *(Pisces, Characidae) Mém Biospéléol 1983 10 171 176

[B37] Abdel-Latif H Hassan ES von Campenhausen C Sensory performance of blind Mexican cave fish after destruction of the canal neuromasts Naturwissenschaften 1990 77 237 239 10.1007/BF01138492 2377235

[B38] Hill LG Feeding and food habits of the spring cavefish, *Chologaster agassizi* Am Midl Nat 1969 82 110 116 10.2307/2423821

[B39] Eigenmann CH Cave Vertebrates of America: a Study in Degenerative Evolution 1909 Washington, DC: The Carnegie Institution of Washington Publications no.104

[B40] Yoshizawa M Jeffery WR Evolutionary tuning of an adaptive behavior requires enhancement of the neuromast sensory system Commun Integr Biol 2011 4 89 91 21509190 10.4161/cib.4.1.14118 PMC3073282

[B41] Yamamoto Y Espinasa L Stock DW Jeffery WR Development and evolution of craniofacial patterning is mediated by eye-dependent and -independent processes in the cavefish *Astyanax* Evol Dev 2003 5 435 446 10.1046/j.1525-142X.2003.03050.x 12950623

[B42] Gross JB Protas M Conrad M Scheid PE Vidal O Jeffery WR Borowsky R Tabin CJ Synteny and candidate gene prediction using an anchored linkage map of *Astyanax mexicanus* Proc Natl Acad Sci USA 2008 105 20106 20111 10.1073/pnas.0806238105 19104060 PMC2629299

[B43] Kirby RF Thompson KW Hubbs C Karyotypic similarities between the Mexican and blind tetras Copeia 1977 1977 578 580 10.2307/1443283

[B44] Cheng R Abney M Palmer AA Skol AD QTLRel: an R Package for genome-wide association studies in which relatedness is a concern BMC Genet 2011 12 66 21794153 10.1186/1471-2156-12-66 PMC3160955

[B45] Broman KW Wu H Sen S Churchill GA R/qtl: QTL mapping in experimental crosses Bioinformatics 2003 19 889 890 10.1093/bioinformatics/btg112 12724300

[B46] Manichaikul A Moon JY Sen S Yandell BS Broman KW A model selection approach for the identification of quantitative trait loci in experimental crosses, allowing epistasis Genetics 2009 181 1077 1086 10.1534/genetics.108.094565 19104078 PMC2651044

[B47] Albertson RC Streelman JT Kocher TD Directional selection has shaped the oral jaws of Lake Malawi cichlid fishes Proc Natl Acad Sci USA 2003 100 5252 5257 10.1073/pnas.0930235100 12704237 PMC154331

[B48] Rapo MA Jiang HS Grosenbaugh MA Coombs S Using computational fluid dynamics to calculate the stimulus to the lateral line of a fish in still water J Exp Biol 2009 212 1494 1505 10.1242/jeb.026732 19411543

[B49] Yoshizawa M Ashida G Jeffery WR Parental genetic effects in a cavefish adaptive behavior explain disparity between nuclear and mitochondrial DNA Evolution 2012 66 2975 2982 10.1111/j.1558-5646.2012.01651.x 22946818 PMC3434958

[B50] Wilkens H Strecker U Convergent evolution of the cavefish *Astyanax *(Characidae, Teleostei): genetic evidence from reduced eye-size and pigmentation Biol J Linn Soc Lond 2003 80 545 554 10.1111/j.1095-8312.2003.00230.x

[B51] Borowsky R Restoring sight in blind cavefish Curr Biol 2008 18 R23 R24 10.1016/j.cub.2007.11.023 18177707

[B52] Hall BK Evolutionary Developmental Biology 1999 2 New York: Springer

[B53] Jones FC Grabherr MG Chan YF Russell P Mauceli E Johnson J Swofford R Pirun M Zody MC White S Birney E Searle S Schmutz J Grimwood J Dickson MC Myers RM Miller CT Summers BR Knecht AK Brady SD Zhang H Pollen AA Howes T Amemiya C Baldwin J Bloom T Jaffe DB Nicol R Wilkinson J Lander ES The genomic basis of adaptive evolution in threespine sticklebacks Nature 2012 484 55 61 10.1038/nature10944 22481358 PMC3322419

[B54] Roberts RB Ser JR Kocher TD Sexual conflict resolved by invasion of a novel sex determiner in Lake Malawi cichlid fishes Science 2009 326 998 1001 10.1126/science.1174705 19797625 PMC3174268

[B55] Yoshizawa M Jeffery WR Shadow response in the blind cavefish *Astyanax *reveals conservation of a functional pineal eye J Exp Biol 2008 211 292 299 10.1242/jeb.012864 18203983 PMC3584714

[B56] Jeffery WR Yamamoto Y The lens is a regulator of craniofacial development and evolution in the teleost *Astyanax* Dev Biol 2000 222 239

[B57] Gallo ND Jeffery WR Evolution of space dependent growth in the teleost *Astyanax mexicanus* PLoS ONE 2012 7 e41443 10.1371/journal.pone.0041443 22870223 PMC3409856

[B58] Nusslein-Volhard C Dahm R Zebrafish: A Practical Approach 2002 New York: Oxford University Press 303

[B59] Rose TM Henikoff JG Henikoff S CODEHOP (COnsensus-DEgenerate Hybrid Oligonucleotide Primer) PCR primer design Nucleic Acids Res 2003 31 3763 3766 10.1093/nar/gkg524 12824413 PMC168931

[B60] Jabado OJ Palacios G Kapoor V Hui J Renwick N Zhai J Briese T Lipkin WI Greene SCPrimer: a rapid comprehensive tool for designing degenerate primers from multiple sequence alignments Nucleic Acids Res 2006 34 6605 6611 10.1093/nar/gkl966 17135211 PMC1747188

[B61] Untergasser A Nijveen H Rao X Bisseling T Geurts R Leunissen JAM Primer3Plus, an enhanced web interface to Primer3 Nucleic Acids Res 2007 35 W71 W74 10.1093/nar/gkm306 17485472 PMC1933133

[B62] Jørgensen JM Coombs S, Görner P, Münz H Evolution of octavolateralis sensory cells The Mechanosensory Lateral Line 1989 New York: Springer-Verlag 115 145

[B63] Manly KF Cudmore RH Meer JM Map Manager QTX, cross-platform software for genetic mapping Mamm Genome 2001 12 930 932 10.1007/s00335-001-1016-3 11707780

[B64] Lincoln SE Lander ES Systematic detection of errors in genetic linkage data Genomics 1992 14 604 610 10.1016/S0888-7543(05)80158-2 1427888

[B65] Broman KW Sen S A Guide to QTL Mapping with R/qtl 2009 1 New York: Springer-Verlag 396

[B66] Lynch M Walsh B Genetics and Analysis of Quantitative Traits 1998 Sunderland, Massachusetts, USA: Sinauer Associates, Inc.

