# Supplementary figures and images for: Evolution of an adaptive behavior and its sensory receptors promotes eye regression in blind cavefish
Source: BMC Biol. 2012 Dec 27;10:108. doi: 10.1186/1741-7007-10-108 (PMC3565949; doi:10.1186/1741-7007-10-108)

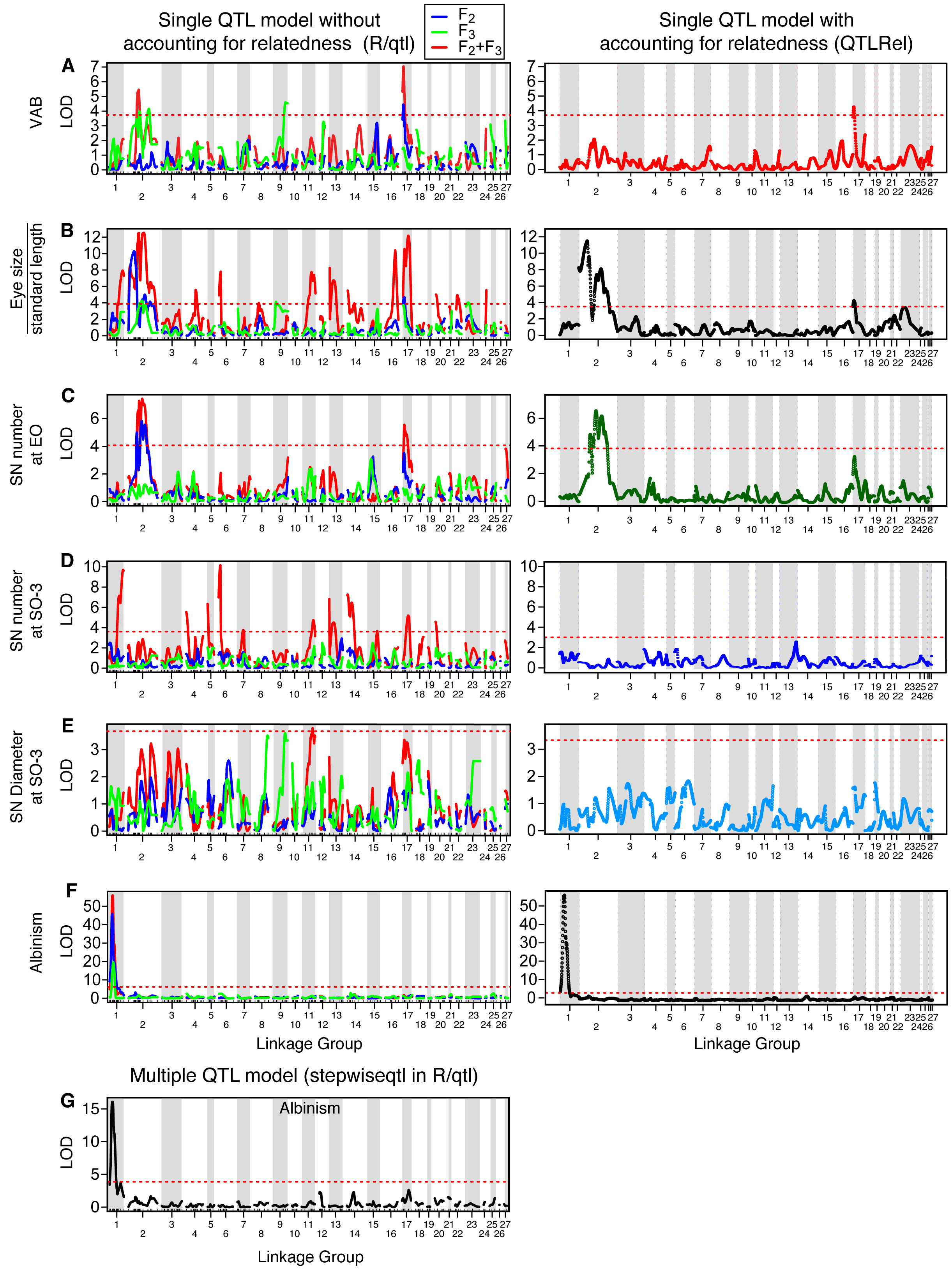

Supplement: Additional file 1 — LOD scores from single-QTL mapping analyses with or without accounting for relatedness among the F2 and F3. (A-F) LOD scores computed with classic single-QTL interval mapping using the F2 dataset (blue lines), F3 dataset (green lines), or F2 + F3 combined dataset (red lines) in R/qtl (left column) and QTLRel (right column). In QTLRel, condense identity coefficients were calculated to adjust background variation according to kinship among the F2 and F3 generations. This analyses found (A) two QTL by the classic scan with the F2 and the F3 dataset (left column), or one QTL (right column) for VAB, (B) five QTL with the F2 and the F3 dataset (left at LG 2, 9, 17 and 23), or three QTL (right at LG2 and 17) for eye-size, (C) one QTL for EO SN number in both, no significant QTL for either (D) SO-3 SN number or (E) SO-3 SN diameter, and (F) one QTL for albinism in the both methods. (G) The LOD score from multiple QTL mapping of albinism also identified a single QTL at the oca2 locus, as described previously [23]. The horizontal lines indicate genome-wide significance of P < 0.05 based on 2,000 permutation tests. [file 1741-7007-10-108-S1.JPEG]

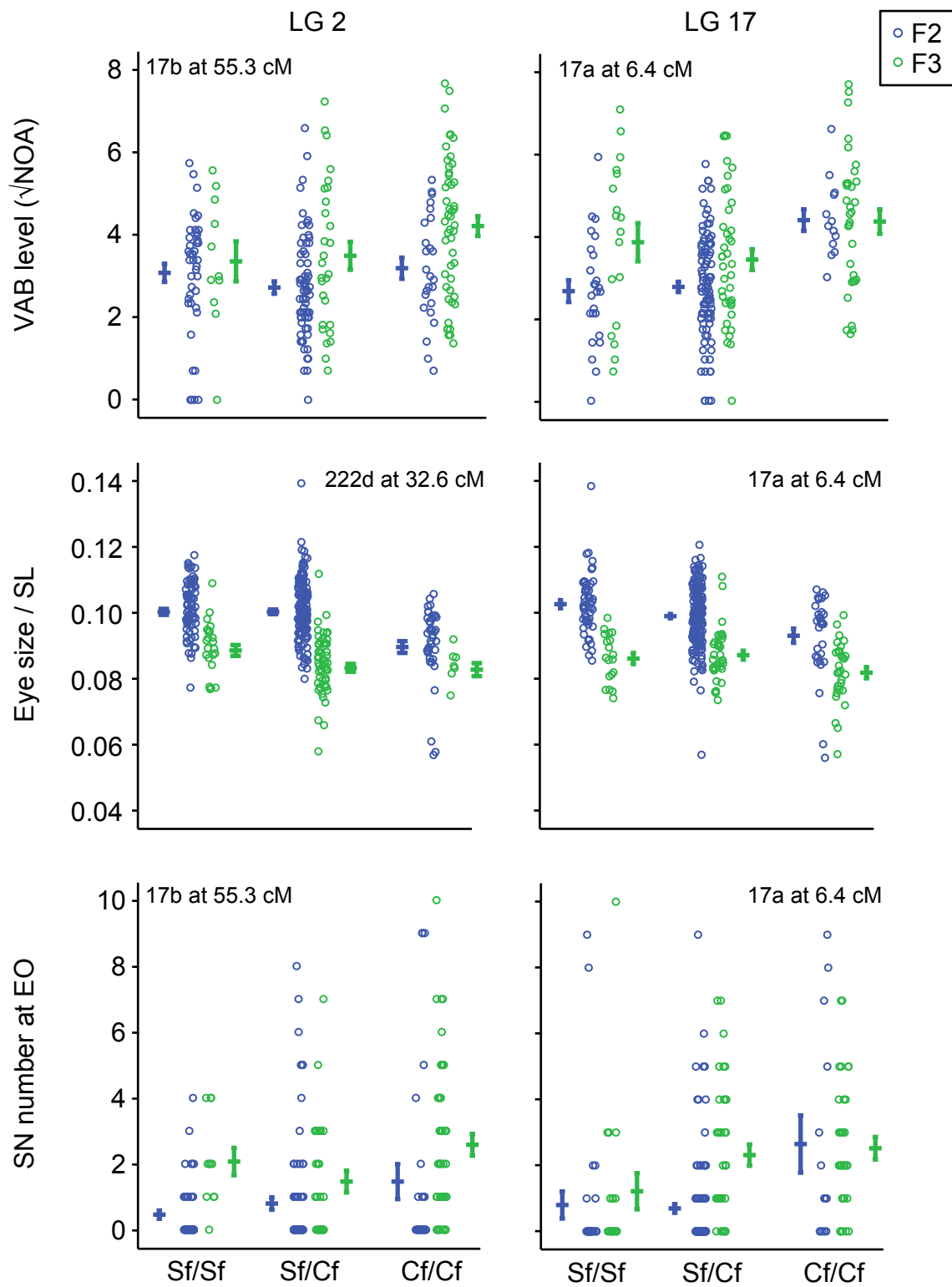

Supplement: Additional file 3 — Scatterplots of phenotypic values against each genotype at the two cluster loci. Scatterplots of phenotypic values of VAB (top row), eye size/SL (middle row) and EO SN number (bottom row) were shown at the marker position of 17b (left column, VAB and EO SN number), 222d (left column, eye size), and 17a (right column, all three traits) at linkage group 2 and 17. Blue dots and bars: F2 generation, Green dots and bars: F3 generation. Bars indicate mean ± s.e.m. Sf/Sf, surface fish homozygote; Sf/Cf, heterozygote; and Cf/Cf, cavefish homozygote. The cavefish alleles at each QTL cluster shift the distributions of VAB, SN and eye size phenotypes in the direction toward higher VAB level, more EO SN and smaller eyes. [file 1741-7007-10-108-S3.PDF]

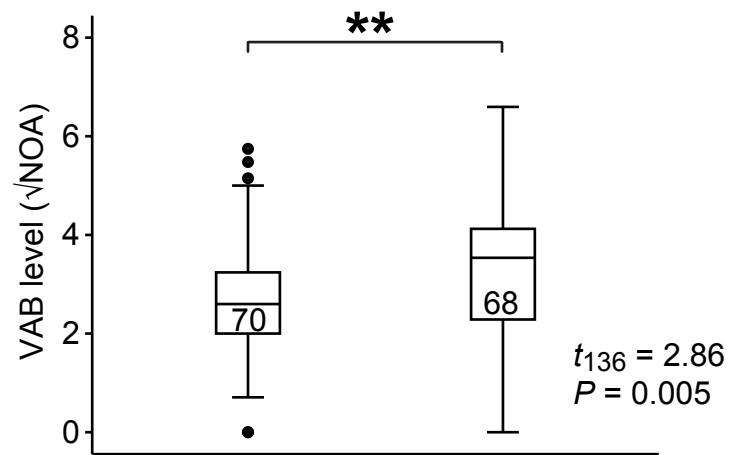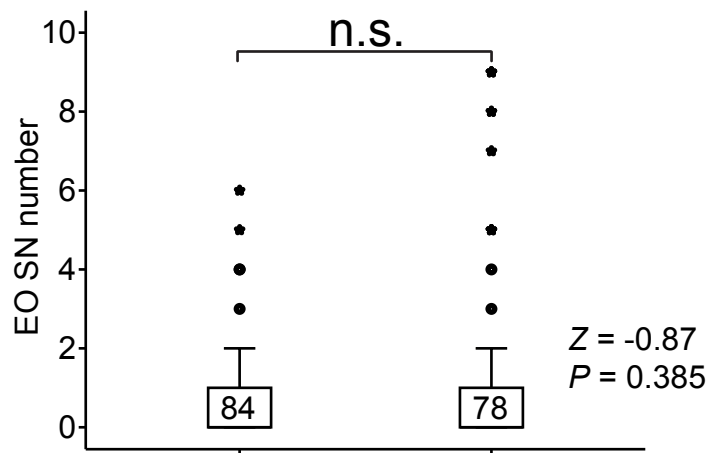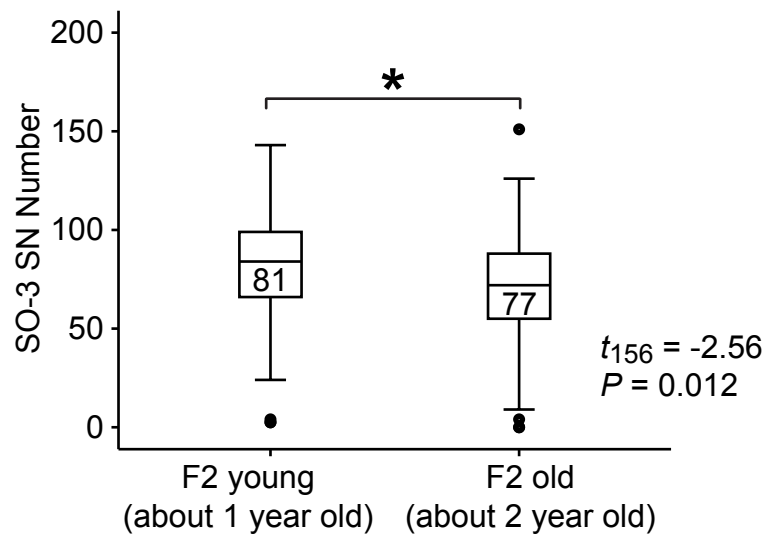

Supplement: Additional file 4 — Phenotypic distributions between approximately one- and two-year-old fish used in current study. The boxplot of phenotypic distributions were compared between approximately one- and two-year-old F2 fish. The increased VAB levels in two-year-old fish (top) was not associated with the increases of EO (middle; no significant difference: n.s.; The Mann-Whitney non-parametric test was applied since phenotypic values were not normally distributed), or SO-3 SN number (bottom; significantly decreased in two-year-old fish). N were indicated in the boxes. [file 1741-7007-10-108-S4.PDF]
