# Supplementary material for: Evolution of an adaptive behavior and its sensory receptors promotes eye regression in blind cavefish
Source: BMC Biol. 2012 Dec 27;10:108. doi: 10.1186/1741-7007-10-108 (PMC3565949; doi:10.1186/1741-7007-10-108)
Supplement: Additional file 2 — Variance Components calculated in QTLRel software. The phenotypes of VAB, Eye size, EO SN number and albinism exhibited small variance components for both the additive and the dominance genetic matrixes, whereas SO-3 SN number and SO-3 SN diameter exhibited large variance components at the dominance genetic matrix. AA: additive genetic matrix; DD: dominance genetic matrix; and EE: the residual matrix. [file 1741-7007-10-108-S2.DOC]

**Variance Components calculated in QTLRel software**

|  | Intercept | AA | DD | EE |
| --- | --- | --- | --- | --- |
| VAB | 3.56 | <0.001 | <0.001 | <0.001 |
| Eye size/ Standard Length | 9.16 | 0.85 | 1.07 | <0.001 |
| EO SN number | 1.53 | <0.001 | <0.001 | 2.04 |
| SO-3 SN number | 98.64 | <0.001 | 1333.51 | <0.001 |
| SO-3 SN diameter | 63.47 | <0.001 | 274.78 | <0.001 |
| Albinism | 0.93 | <0.00001 | <0.00001 | 0.0624 |

AA: additive genetic matrix; DD: dominance genetic matrix; and EE: the residual matrix.
