# Supplementary material for: Evolution of an adaptive behavior and its sensory receptors promotes eye regression in blind cavefish
Source: BMC Biol. 2012 Dec 27;10:108. doi: 10.1186/1741-7007-10-108 (PMC3565949; doi:10.1186/1741-7007-10-108)
Supplement: Additional file 5 — Candidate gene primer and probe sets used in this study. Primers for the microsatellite markers were designed to amplify 150 to 350 base-pair genomic fragments containing length polymorphisms between surface fish and cavefish. Primers and fluorescent probes for Taqman and HybProbe genotyping methods were designed to detect single nucleotide polymorphisms between these two morphs. [file 1741-7007-10-108-S5.PDF]

Candidate gene primer and probe sets used in this study

Microsatellite Markers

Gene name	**Forward Primer**	**Reverse Primer**
cryaa	GGGAGGCTGCAGAGTACTGA	CATACAGCCAGAGGCAGACA
eya1	CCCTAAGTCAGGAATTCAGTCTTCT	TCTGTTTGGCTCATCTCAACAT
mao	AGGCTGTACTTTGCTGGTA	ATCTTTTTAAATGGACAACTTG
mchr2	AAACAGCAAAATCAGAGAAACTGA	ACCAGGGTTATTCCACCAAA
ngn1	CGTTTTCGTTTGACATTGCT	GCTGGAGTGGGGTCTGTAAA
nrg2	GTCGCAGTCTCGGTCCAAA	CTTATAGGTGCAGGTCTATAGCATATC
pmch	CCAACACACACCACCAAACT	CTGCTTGAGACTAAACCCATGT
pfn2	TGTCTGCAAAAGTTGTCAAA	CTACATGGTCGGCTTAGGTT
sert	ACTGCCATGTCTAAATCTG	CATGTAGCCCAGCACAGTGA
th	TGTGGCACAGCAACAAGTG	TCTCCAGCAGCTTCAATGC
tph2	CTACGGCTCTTTCAGGTCAGTA	AGGCAGAACACACCCTATGTAT

Taqman Primer and Probes

Gene name	**Forward Primer**	**Reverse Primer**	**Probe for surface fish allele**	**Probe for cavefish allele**
5ht1A	AGGTTGGGAAAGCGATGCT	ACCTGCTGTTTTTATTGGGTTTTT	CAGAGAAAAACTGAAGAAAA	CAGAGAAAAACTGAAGAAGA
5ht2A	ATGTGGTGCCCGTTCTTTG	AATCATTAGTGAAGGGTCACAAGCT	CTGGTGGTATGCGAC	CTGGTGGTATGCAAC
5ht2B	ACAGCTGCAGCCCAAGTGT	TGTAATTGAGGACTGAAAACAGGAAA	CACATGTGGATAAATG	CACATGTGGATAAATT
5ht2C	TCTATGAGACCAAGGCAT	GTGAGTTAAGCTGAGACG	N/A	CTCAAAGAGTTCCCGTCCCC
5ht2C_like	CAGTAGTGTCTGATTGATGGAACCA	CAATTAGAAGAAGAGCAGGAAAAATACA	AGGACTGGGAGTCT	CAGGACTGGGATCC
shroom2	CAGATTGTGGTCCTCAGATAAGCA	CAGCTCAAGTGTCTGATGGACAGT	TCTGGTCTGGAGGCA	CTCTGGTCTGGAAGC
th2	GCAGTGTACTCAACACACGGTAGTG	TTCAACTACAAACAGTGAGTACCAATCA	CTGTGAAACATGTAAAG	CTGTGAAACATGTCAAGA

HybProbe and Primers used under Roche Light Cycler

Gene name	**Forward Primer**	**Reverse Primer**	**Probe1**	**Probe2**
drd1	TTCTCACTGGCTGCTTTC	CAAGGCATGACCAGGAT	ACGCTGGTCTGTGCGG-Fluorescein	LC Red 640-GTCACCAAATTTCGCCACCTCCGC-Phosphate
mchr1	TTACAGTGGAACATAGAACACATATC	TAGGTGTTTAAACTGTAGACTGG	AGGGCCCGAAGATCAAGAGCATTTAA-Fluorescein	LC Red 640-TTGGGAAATGGGGGAAATGGCAAA-Phosphate

Note: Genomic markers for cryaa, shroom2 and mchr1 were newly designed in this study.
